# The Impact of Thrombocytopenia on Outcome in Patients with Acute Coronary Syndromes: A Single Center Retrospective Study

**DOI:** 10.1155/2015/907304

**Published:** 2015-10-04

**Authors:** Andreja Sinkovič, Maja Majal

**Affiliations:** ^1^Department of Medical Intensive Care, University Clinical Center Maribor, Ljubljanska 5, SI-2000 Maribor, Slovenia; ^2^Department of Hematology, University Clinical Center Maribor, Ljubljanska 5, SI-2000 Maribor, Slovenia

## Abstract

*Background*. In acute coronary syndromes (ACS), treated by combined antithrombotic therapy and percutaneous coronary interventions (PCI), thrombocytopenia may occur. Our aim was to evaluate predictors and the impact of thrombocytopenia on mortality in high-risk ACS patients. *Methods*. We retrospectively evaluated high-risk ACS patients. Thrombocytopenia was defined as platelet count <140.000/mL or a drop in platelet count of >50% during in-hospital stay. We compared demographic, laboratory, clinical, and mortality data between nonthrombocytopenic and thrombocytopenic ACS patients and evaluated independent predictors of thrombocytopenia. *Results*. In 371 ACS patients, thrombocytopenia was observed in 21.3%. Thrombocytopenic patients were significantly older and, less likely treated by PCIs (72.1% versus 89.7%, *p* < 0.001) and combined antithrombotic therapy, with increased incidence of in-hospital complications and the use of additional treatments, but with increased mortality at 30 days (27.8% versus 10.2%, *p* < 0.001) and 6 months (35.4% versus 13.6%, *p* < 0.001) when compared to nonthrombocytopenic patients. The use of antibiotics, transfusions, insertion of intra-aortic balloon pump (IABP), and prior stroke independently predicted thrombocytopenia. *Conclusions*. Thrombocytopenia, observed in about 20% of high-risk ACS patients, was associated significantly with in-hospital complications and mortality. Predictors of thrombocytopenia were the use of antibiotics, transfusions, insertion of IABP, and prior stroke.

## 1. Introduction

Acute coronary syndromes (ACS) with (STEMI) or without (NSTEMI) ST-segment elevation are mainly the consequence of acute coronary atherothrombosis [1]. The most effective therapy is percutaneous coronary intervention (PCI), either primary PCI in STEMI or early PCI in high-risk NSTEMI patients, resulting in coronary recanalization and myocardial reperfusion [2, 3].

PCI in STEMI and high-risk NSTEMI patients should be accompanied by combination of antiplatelet and anticoagulant drugs to prevent thrombus formation at the site of coronary intervention [2, 3]. Antiaggregatory effect of antiplatelet drugs together with pronounced anticoagulant effect of heparins, either unfractionated or of low molecular weight, can also lead to increased risk of bleeding and increasing mortality in high-risk ACS patients [1, 4]. Platelet dysfunction can be particularly expressed within the first hours after PCIs, related with the loading doses of antiplatelet agents. If this leads to clinical thrombocytopenia, mortality in ACS patients may exceed 20% [5].

Thrombocytopenia is defined mostly as a decrease of platelet count below referenced lower limit of normal or a drop in platelet count of more than 50% during in-hospital stay [6]. A low platelet count in high-risk ACS patients can be the consequence of different causes. It may be either immunomediated due to heparins, glycoprotein receptor IIb/IIIa (GPIIb/IIIa) inhibitors, or thienopyridines or consumptional, due to PCI or insertion of intra-aortic balloon pump (IABP) or due to acute heart failure [7]. In the GRACE registry, 0.3% of thrombocythemia was heparin-induced (HIT), 0.6% was glycoprotein-associated (GAT), and 0.7% was of other origins [8]. Profound thrombocytopenia (<100.000) was observed in 2.5% of myocardial infarcts after abciximab and 0.5% after tirofiban treatment combined with PCI and dual oral antiplatelet agents and in 0.6% of patients treated by GPIIb/IIIa inhibitors according to the GRACE registry [8–10]. Clopidogrel-related thrombocytopenia was observed in 1.0% patients after percutaneous stent implantation [11]. After PCI, about 16% of patients developed a moderate to severe decline in platelet count according to De Labriolle et al. [12].

After the use of the direct thrombin inhibitor bivalirudin, in comparison to heparin with GPIIb/IIIa inhibitor, thrombocytopenia developed in 3.7% [13].

Acute heart failure in high-risk ACS patients, as the consequence of myocardial dysfunction due to extensive ischemic necrosis, promotes neurohumoral activation, inflammation, and acute kidney injury. Activation of inflammation in acute heart failure seems an important stimulus for thrombocytopenia due to enhanced platelet clearance by macrophages [14]. Also, hypotension in severe acute heart failure may inhibit production of platelets in the bone marrow [15]. In patients with IABP, thrombocytopenia can develop even in 43–58%, according to one study [16].

In NSTEMI patients, low platelet count was independently associated with female sex, ST-segment depression, PCI, heart failure, systolic blood pressure, heart rate, and so forth, in one cohort [5]. However, De Labriolle et al. demonstrated that thrombocytopenia in coronary patients after PCI was predicted by male gender, age, hypercholesterolemia, acute renal and heart failure, IABP insertion, STEMI, reduced hematocrit, heparin use, and low osmolar contrast agent [12]. Therefore, these studies support a possibility of a multicomponent background for decreased platelet counts [5, 12, 16].

The incidence of thrombocytopenia varies in different trials. In the CRUSADE registry, 13% of NSTEMI patients, in ACUITY trial, only 6.8% of ACS patients, and in the GRACE registry, 1.6% of STEMI or NSTEMI patients developed reduced platelet counts [4, 5, 8].

These registries have found increased risk of mortality if thrombocytopenia was observed in the setting of ACS [4, 5, 8]. In the GRACE registry, thrombocytopenia was associated with up to 21% in-hospital mortality [8]. In ACUITY trial, 30-day and 1-year mortality correlated with severity of thrombocytopenia, being approximately 9% at 30 days and at one year in severe decline in platelet count [4].

Our aim was to evaluate the incidence of thrombocytopenia in every-day clinical practice in high-risk ACS patients, STEMI, and high-risk NSTEMI patients and the impact of low platelet count on treatments, in particular on the use of PCIs, on in-hospital complications (bleeding, reinfarctions, heart failure, and acute renal failure) and mortality (within 30 days and 6 months), and on predictors of thrombocytopenia.

## 2. Methods

### 2.1. Study Design

This was a retrospective observational study, approved by the National Medical Ethics Committee of the Republic of Slovenia (number 123/06/10), who waived the need for informed consent because of the retrospective nature of the study. Personal data of all the patients were protected according to the Law on Personal Data Protection.

### 2.2. Patients and Protocol

We analysed relevant data of patients being admitted to the Medical ICU of the University Clinical Centre of Maribor with acute myocardial infarction as a discharge diagnosis. We obtained a list of 371 consecutive ACS patients through the institutional medical information system, meeting either standard criteria for STEMI or high-risk NSTEMI and recorded their clinical, demographic, and mortality data [2, 3]. As soon as STEMI/NSTEMI was diagnosed by emergency medical services, the patients received antithrombotic therapy at the first medical contact according to the current ESC guidelines (acetylsalicylic acid (ASA), clopidogrel, and heparin i.v.), oxygen to maintain SatO_2_ 94–98%, morphine with antiemetics, and sublingual nitroglycerin if deemed necessary [2]. In case of pulmonary edema and/or cardiogenic shock, these ACS patients were treated by morphine and/or diuretics and vasopressors and/or were intubated and mechanically ventilated if deemed necessary. All patients in cardiac arrest were resuscitated appropriately and those who had successful cardiopulmonary resuscitation (CPR) for ventricular fibrillation (primary VF-arrest) subsequently underwent angiography with a view of intervention as all STEMI patients [2].

The high-risk NSTEMI patients usually received antithrombotic therapy according to the ESC guidelines after admission to medical ICU if there were no contraindications for their use (ASA, clopidogrel, and unfractionated or low-molecular-weight heparin). Angiography and PCI were performed within the first hours of ICU stay [3]. The first blood samples were usually obtained just before or after the PCI for routine tests. After PCI, either primary (STEMI) or early emergency (NSTEMI), noninvasive monitoring (continuous ECG and pulse oximetry, blood pressure measurements hourly) was initiated for at least the first 24 hours. Standard ECG and basic laboratory parameters had been repeated after intervention, including Troponin I [2, 3].

Within the first few hours after PCI, the patients usually received oxygen by face mask or by nasal prongs, i.v. infusion of fluids to prevent renal injury, and i.v. infusion of an GPIIb/IIIa inhibitor at the discretion of the treating physician to prevent in-stent thrombosis [2, 3]. The GPIIb/IIIa inhibitor was usually discontinued 12 or 24 hours after the PCI according to the current ESC guidelines [2, 3].

Statins, beta blockers, and angiotensin-converting enzyme inhibitors were prescribed after the first 24 hours. Dual antiplatelet therapy (ASA and clopidogrel) in maintenance dose was continued to prevent in-stent thrombosis and progression of ischemia [2, 3].

Standard ECG and laboratory tests were usually repeated on daily basis and an echocardiography at least once during in-hospital stay. Echocardiography and other diagnostic procedures (chest X-ray, coronary angiography) were repeated in suspected complications [2, 3].

Acute myocardial infarction (MI) was diagnosed with the rise and fall of Troponin I in addition to ECG changes with or without Q waves [2, 3].

We registered demographic and clinical data on admission and during in-hospital stay, in-hospital treatments and mortality data (at 30 days and 6 months).

Regarding demographic data, we registered age, gender, comorbidities (arterial hypertension, diabetes, dyslipidemia, prior myocardial infarction, and stroke), and smoking. From laboratory data, we registered platelet count and Troponin I levels on admission and peak levels during the hospital stay.

Among in-hospital treatments, we registered PCIs and the use of antithrombotic therapy (ASA clopidogrel, heparins, and GPIIb/IIIa inhibitors).

In terms of in-hospital complications, we registered acute heart failure, arrhythmias, reinfarctions, bleeding, and acute kidney injury at any time of in-hospital stay. Arrhythmias, registered by continuous ECG monitoring and standard ECG recordings, were defined as atrial or ventricular or conduction disturbances [2].

Heart failure was quantified by the Killip classification as classes II–IV, including patients with pulmonary congestion, pulmonary edema, and cardiogenic shock [2, 3].

Reinfarctions were classified as recurrence of chest pain with new ECG changes and recurrent rise and fall of serum Troponin I [2].

Bleeding was considered major (cerebral or symptomatic bleeding of other locations with a drop in hemoglobin >50 g/L or the need of ≥2 units of blood product transfusions) or minor (symptomatic bleeding with a drop in hemoglobin of 30–50 g/L) or minimal (symptomatic bleeding with a drop in hemoglobin <30 g/L), according to TIMI criteria [2, 3, 17].

Acute kidney injury was defined as an increase of serum creatinine of more than 50% within 48–72 hours, according to Acute Kidney Injury Network (AKIN) criteria [18, 19].

In case of complications, the patients were treated according to professional protocols (e.g., by vasopressors, inotropic agents, mechanical ventilation, intra-aortic balloon pump (IABP), red blood cells transfusions, antibiotics, antiarrhythmic drugs, and pacing) [2, 3].

### 2.3. Laboratory Tests

Troponin I levels were determined by the immunochemical method (Boehringer, Germany, normal levels < 0.015 *μ*g/L) [20].

C-reactive protein (CRP), a marker of inflammation, was measured by immunochemical method (Siemens Healthcare Diagnostics, Germany; normal levels were <3.0 mg/L).

Platelet count was measured by the Sysmex XE2100 automatic analyser, Kobe, Japan (normal levels 140.000/mL–340.000/mL) [6]. Thrombocytopenia in ACS patients was defined as platelet count <140.000/mL or a drop in platelet count >50% during in-hospital stay, including patients with admission platelet count <140.000/mL [6].

### 2.4. Statistical Analysis

Statistical analyses were performed using the SPSS statistical package, version 19 (SPSS Inc., Chicago, IL, USA) for Windows. Data were expressed as mean ± standard deviations or percentages. Differences between the groups were tested by the two-sided Student's *t*-test for mean ± standard deviations and by the chi-square test for percentages. A *p* value < 0.05 was considered statistically significant. To identify independent predictors of thrombocytopenia, all significant variables of interest gained by univariate analysis were entered in a model of binary logistic regression.

## 3. Results

**Figure 1:**
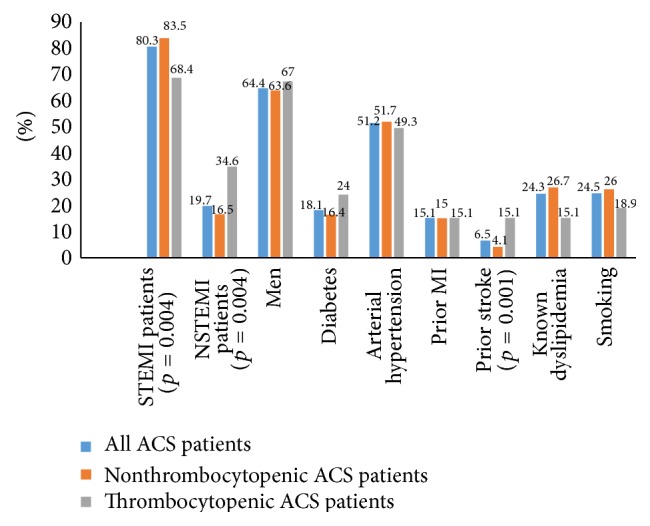
Baseline clinical data in all ACS patients and in nonthrombocytopenic and thrombocytopenic ACS patients. ACS, acute coronary syndrome; STEMI, ST-elevation myocardial infarction; NSTEMI, non-ST-elevation myocardial infarction; MI, myocardial infarction.

Thrombocytopenia was observed in 21.3% of all ACS patients. Baseline characteristics of our ACS patients and differences between nonthrombocytopenic and thrombocytopenic patients are displayed in Table 1 and Figure 1.

Between the nonthrombocytopenic and thrombocytopenic patients, we observed statistically significant differences in mean age (63.3 ± 12.8 years versus 66.8 ± 11.8 years, *p* = 0.032), mean admission CRP (16.2 ± 42.3 mg/L versus 28.8 ± 53.0 mg/L, *p* = 0.030), and peak CRP levels (66.0 ± 82.8 mg/L versus 117.6 ± 93.8 mg/L, *p* < 0.001), prior stroke (4.1% versus 15.1%, *p* = 0.001), the incidence of STEMI (83.5% versus 68.4%, *p* = 0.004), and NSTEMI (16.5% versus 34.6%, *p* = 0.004) (Figure 1) (Table 1).

**Figure 2:**
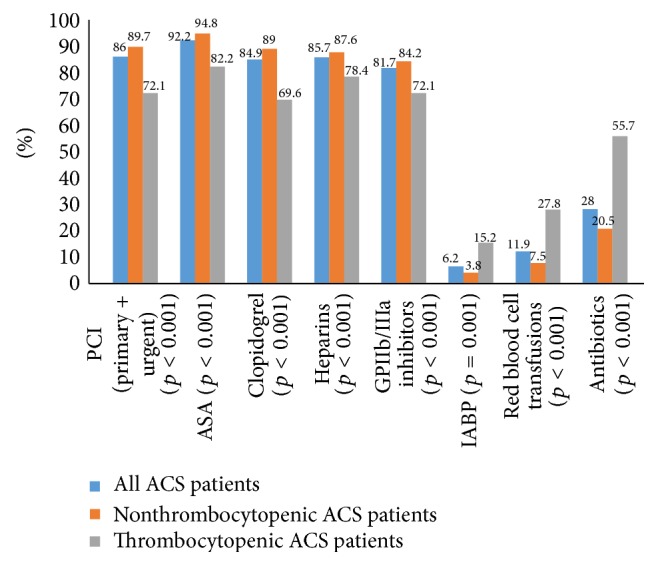
In-hospital treatment of all ACS patients and nonthrombocytopenic and thrombocytopenic ACS patients. ACS, acute coronary syndromes; PCI, percutaneous coronary intervention; ASA, acetylsalicylic acid; GPIIb/IIIa, glycoprotein receptor IIb/IIIa; IABP, intra-aortic balloon pump.

Treatments for our ACS patients are presented in Figure 2.

PCIs had been performed significantly less often in thrombocytopenic patients (72.1% versus 89.7%, *p* < 0.001) and antithrombotic therapy was used less frequently than its use in nonthrombocytopenic patients (ASA 82.2% versus 94.8%, *p* < 0.001; clopidogrel 69.6% versus 89%, *p* < 0.001; heparins 78.4% versus 87.6%, *p* = 0.024; GPIIb/IIIa antagonists 72.1% versus 84.2%, *p* = 0.027) (Figure 2).

**Figure 3:**
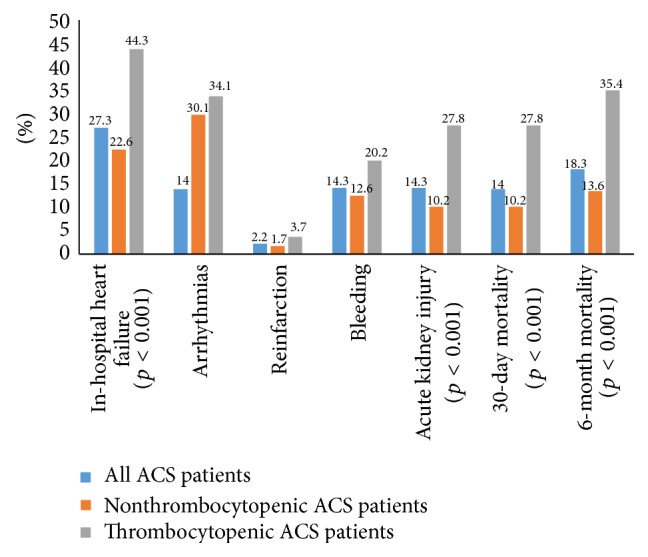
In-hospital complications and mortalities in all ACS patients and thrombocytopenic and nonthrombocytopenic ACS patients. ACS, acute coronary syndromes.

In-hospital complications and mortality data are presented in Figure 3.

In-hospital complications, such as heart failure, were more frequent in thrombocytopenic patients (44.3% versus 22.6, *p* < 0.001) and acute renal failure (27.8% versus 10.2%, *p* < 0.001) was observed significantly more often (Figure 3), as well as treatment by IABP (15.3% versus 3.8%, *p* = 0.001), red blood cell transfusions (27.8% versus 7.5%, *p* < 0.001), and use of antibiotics (55.7% versus 20.5%, *p* < 0.001) than in the nonthrombocytopenic patients (Figure 2). We observed significant increase in 30-day (27.8% versus 10.2%, *p* < 0.001) and six-month mortality (35.4% versus 13.6%, *p* < 0.001) in thrombocytopenic ACS patients in comparison to those with normal platelet count (Figure 3).

Severe thrombocytopenia (<50.000/mL) was observed only in 3 patients; one of them died within 30 days. Moderate thrombocytopenia (<100.000/mL) was observed in 15 patients. Six of them died within 30 days. Mild thrombocytopenia (platelet count < 140.000/mL and >100.000/mL) was observed in 64 ACS patients. Sixteen of them died within 30 days.

Significant variables of interest gained by univariate analysis were entered in a model of binary logistic regression, which found significant independent predictors of thrombocytopenia during the in-hospital stay as insertion of IABP, prior stroke, the use of antibiotics, and red blood cell transfusion (Table 2).

## 4. Discussion

In-hospital thrombocytopenia, being mostly mild and transient, was observed in 21.3% of our high-risk ACS patients and was associated with less frequently performed PCIs and with more than 2-fold increased 30-day and six-month mortality. Post-PCI treatments of complications by IABP insertion, red blood cell transfusion, and use of antibiotics as well as prior stroke significantly and independently predicted thrombocytopenia during the in-hospital stay.

Coronary revascularization by PCIs in high-risk ACS patients should be combined with antithrombotics for prevention of coronary reocclusion at the site of coronary intervention [2, 3]. Antithrombotics prevent residual platelet reactivity and thus coronary thrombosis but can trigger thrombocytopenia [7, 8, 12]. Drug-induced thrombocytopenia in a setting of ACS is difficult to affirm as there are no specific tests, except for HIT [7]. In addition, multiple causes for thrombocytopenia coexist, including pharmacological, procedural, and clinical ones such as complications of the acute coronary event [7].

In the ACS setting, there are two main types of drug-induced thrombocytopenia, that is, HIT and GIT, with a different prognosis for each type. Mild and transient decline in platelet count occurring 1–4 days after initiation of therapy is common and observed in up to 15% of unfractionated heparin-treated patients. It is not immunomediated and rarely leads to a severe reduction in platelet levels. It mostly resolves spontaneously, despite continuation of unfractionated heparin [7, 8, 12]. The majority of ACS patients in this cohort with reduced platelet count belonged to this type of thrombocytopenia.

In our ACS patients, the reversible small molecular GPIIb/IIIa inhibitor, eptifibatide, was used predominantly and abciximab was used only in individual cases. The time interval from the administration of i.v. eptifibatide to the onset of thrombocytopenia was 48–72 hours, suggesting the possibility of eptifibatide as the cause of thrombocytopenia, but that was not proven as specific tests are lacking [10, 21].

Heparins, either nonfractionated or of low molecular weight, are an important potential cause of immunomediated thrombocytopenia, HIT. In our ACS patients, HIT antibodies were demonstrated only in 1 patient, who was exposed to heparin in prior hospitalization. Thrombocytopenia developed within the first few days of in-hospital stay in the majority of our patients, mostly within the first 5 days in contrast to HIT, which generally occurs between 5 and 10 days [7, 8, 12].

On the other hand, PCIs were performed less often in ACS patients with reduced platelet count; therefore, antithrombotic drugs were also given less frequently to them than to patients with normal platelet counts.

PCIs were not performed in those presenting late with STEMI or those with complex multivessel disease in a setting of either NSTEMI or STEMI, if early death occurred or in those who refused the interventional therapy. Regarding complex multivessel coronary disease, current ESC STEMI guidelines recommend strategy of “culprit vessel only” primary PCI, followed by further elective revascularisation in case of ongoing ischaemia. This can lead to a substantial delay in reperfusion of the myocardium at risk [2]. This problem was in particular evident in STEMI patients with apparently new left bundle branch block. The advantage of complete revascularization at the time of presentation for STEMI patients over “culprit vessel only” strategy was demonstrated by the recent Culprit trial but needs further evaluation [22].

In addition, radial arterial access for PCI has been proven to offer an advantage in ACS setting with increased risk of bleeding by decreasing the risk of vascular complications and mortality within 30 days when compared to femoral arterial access [2].

The main reasons not to use antithrombotic agents were early complications such as early death and renal failure, as well as early CABG. However, PCIs and antithrombotic agents were still used in approximately 70% of our thrombocytopenic ACS patients.

When early coronary revascularization is delayed or abandoned in the setting of ACS, extensive myocardial necrosis develops, which can be followed by severe myocardial dysfunction with subsequent activation of neurohumoral factors and of inflammation to lead to increased platelet consumption and thrombocytopenia. Thus, thrombocytopenia may act as a marker of the acuity and severity of the inflammation in ACS settings. In addition, activated inflammation and neurohumoral system can further impair hemodynamics and contribute to other organ hypoperfusion, including that of the kidneys [2, 3, 7, 12]. In our thrombocytopenic ACS patients, we observed increased incidence of acute heart failure and of kidney injury, increased levels of the inflammatory marker CRP and, finally, increased short- and long-term mortality.

Several trials demonstrated that thrombocytopenia in addition to drug-induced antiplatelet effect in ACS patients was associated with increased risk of bleeding. However, bleeding was not significantly increased in our thrombocytopenic patients, but red blood cells were significantly more often transfused than in the nonthrombocytopenic patients. Red blood cells were transfused in case of major bleeding, but also in case of severe anemia with hemoglobin level < 80 g/L irrespective of etiology to improve oxygen delivery in the setting of coronary ischemia.

No platelets were transfused, what is in accordance with current guidelines, which recommend infusion of platelet rich plasma only in pronounced drop in platelet count (<10.000/mL) in case of active bleeding [2, 3, 7]. We observed a drop in platelet count below 50.000/mL only in 3 patients (0.8% of all ACS patients) and the platelet count dropped below 100.000/mL only in 15 patients (4% of ACS patients). Other studies observed a drop of platelets below 50.000/mL in approximately 6% of ACS patients, treated by combination of antiplatelet and anticoagulant agents [4, 5].

We observed that even a mild drop in platelet count in high-risk ACS patients was associated with increased mortality and complication rate, what is consistent with the results of other studies [4, 5, 8, 12, 23, 24]. The short- and long-term mortality were twofold increased in our thrombocytopenic ACS patients as compared to those with normal platelet counts. In addition, treatment modalities such as insertion of IABP, use of antibiotics, and blood transfusions predisposed patients to thrombocytopenia even more. Our data confirm that thrombocytopenia in high-risk ACS patients seems to be multifactorial. It may be associated with drug effects such as GPIIa/IIIb inhibitors, heparins, and even clopidogrel, but also procedures such as PCIs and IABP insertion. An important predisposing factor in ACS setting is myocardial pump failure with organ hypoperfusion after delayed percutaneous revascularization, which can be aggravated by activated neurohumoral system and inflammation. Acute kidney injury is an important complication of tissue hypoperfusion, but it can also be induced by contrast agents during PCI [4, 5, 7, 8]. In our ACS patients, acute kidney injury was significantly more frequent in thrombocytopenic patients, in particular, in combination with heart failure [2, 3, 18, 19].

In recent years, novel antiplatelet and anticoagulant drugs have been developed and tested. Their use seems promising in preventing drug-induced thrombocytopenia. Novel ADP-receptor antagonists, like prasugrel and ticagrelor, may also induce thrombocytopenia with earlier onset of action than clopidogrel. It appears prudent to replace the heparin plus GPIIb/IIIa inhibitor regime by one of the novel anticoagulants such as bivalirudin in STEMI or fondaparinux in NSTEMI patients presenting with low platelet counts [2, 25]. This strategy may help to reduce the risk of drug-induced thrombocytopenia to some extent [2, 3, 7].

### 4.1. Conclusions

Our conclusions are as follows: even mild degree of thrombocytopenia, observed in approximately 20% of high-risk ACS patients, is significantly associated with less often performed PCIs and with several complications, including increased short- and long-term mortality. However, thrombocytopenia was independently predicted by post-PCI procedures, aimed at mastering in-hospital complications. Therefore, early treatment of high-risk ACS patients should focus on early PCI to prevent large ischemic necrosis: particularly in the elderly with comorbidities and in patients with hemodynamic instability or with large anterior infarcts, which can reduce mortality (the higher-risk patients benefit most from early PCI strategies). The use of novel antiplatelets with reversible platelet inhibition (ticagrelor) and antithrombotic drugs with direct thrombin inhibition (bivalirudin) can reduce the risk of drug-induced thrombocytopenias during interventional therapy and should be preferred.

Regarding PCI procedures multivessel coronary interventions at presentation should be performed in case of severe acute heart failure to prevent delays in reperfusion in particular in STEMI setting. To avoid any further bleeding risks, radial vascular approach has the priority if possible.

### 4.2. Limitation of the Study

Our study is a retrospective observational design with limited number of patients, carrying all the limitations and biases inherent in such studies. However, the data are taken from the “real world” as an observational study and therefore can reflect on the complexity of managing ACS patients with thrombocytopenia during our daily practice.

## Figures and Tables

**Table 1:** Clinical and laboratory data of all ACS patients and nonthrombocytopenic and thrombocytopenic ACS patients.

Clinical and laboratory data (mean ± SD)	All (*n* = 371)	Nonthrombocytopenic (*n* = 292)	Thrombocytopenic (*n* = 79)	*p* values
Age (years)	64.1 ± 12.7	63.3 ± 12.8	66.8 ± 11.8	0.032
Admission of Troponin I (*μ*g/L)	11.4 ± 23.5	10.6 ± 22.4	14.3 ± 27	ns
Peak Troponin I (*μ*g/L)	41.5 ± 36.2	41.5 ± 35.6	41.3 ± 38.7	ns
Admission CRP (mg/L)	18.9 ± 45.0	16.2 ± 42.3	28.8 ± 53.0	0.030
Peak CRP (mg/L)	78.1 ± 88.1	66.0 ± 82.8	117.6 ± 93.8	<0.001
ICU stay (days)	3.6 ± 4	2.9 ± 2.3	6.0 ± 6.9	ns
In-hospital stay (days)	10.2 ± 22.7	9.6 ± 25.0	12.4 ± 10.5	<0.001

ACS, acute coronary syndrome; SD, standard deviation; CRP, C-reactive protein.

**Table 2:** Binary logistic regression model to identify independent predictors of thrombocytopenia in ACS patients.

Variables	OR	95% confidence interval	*p* values
PCIs	2.362	0.695 to 8.033	0.169
ASA treatment	0.633	0.138 to 2.900	0.556
Clopidogrel treatment	1.739	0.543 to 5.577	0.352
Heparin treatment	1.700	0.690 to 4.185	0.249
GPIIb/IIIa inhibitors	0.531	0.185 to 1.528	0.241
Antibiotics	0.405	0.189 to 0.866	0.020
Red blood cell transfusion	0.371	0.143 to 0.963	0.042
Acute heart failure	0.822	0.395 to 1.713	0.601
Acute kidney injury	1.448	0.559 to 3.750	0.446
Stroke	0.292	0.101 to 0.848	0.024
IABP	0.232	0.620 to 0.871	0.030
STEMI	1.388	0.630 to 3.057	0.415
Age	0.995	0.970 to 1.0190	0.671

PCI, percutaneous coronary intervention; ASA, acetylsalicylic acid; GPIIb/IIIa, glycoprotein receptor IIb/IIIa; IABP, intra-aortic balloon pump; STEMI, ST-elevation myocardial infarction; OR, odds ratio.
